# Supplementary material for: Detecting phenotype-driven transitions in regulatory network structure
Source: NPJ Syst Biol Appl. 2018 Apr 19;4:16. doi: 10.1038/s41540-018-0052-5 (PMC5908977; doi:10.1038/s41540-018-0052-5)
Supplement: Supplementary file 1 — Supplementary Figures [file 41540_2018_52_MOESM1_ESM.pdf]

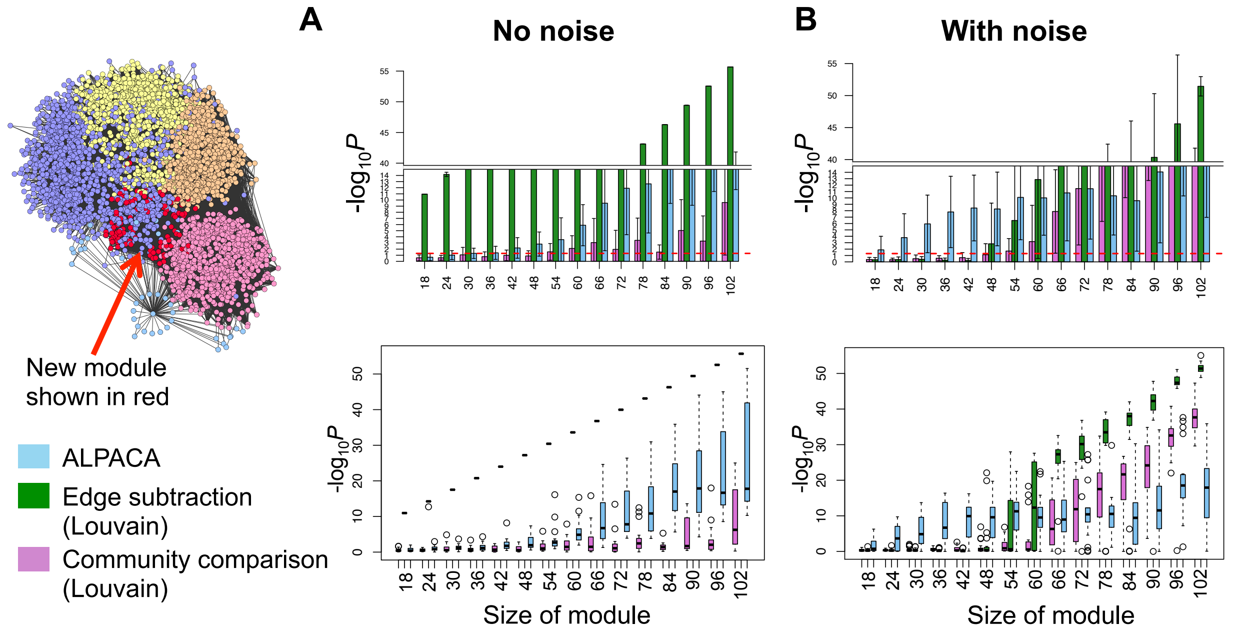

Supplementary Figure 1. Performance of three Louvain-based differential community methods on simulated networks with added module. Network at left visualizes the regulatory network derived from normal human fibroblasts (as shown in Figure 2). Bar graphs show performance of each method – ALPACA, edge subtraction with Louvain optimization, or community comparison with Louvain optimization – on network simulations with (**A**) or without (**B**) resampling of edges among the pre-existing communities. P-values computed using a one-sided Wilcoxon test. Bar graphs show mean of $-log_{10}P$ over twenty network simulations, and error bars depict the corresponding standard deviation. Boxplots represent same data as bar plots. Boxplot elements are defined as follows: center line, median; box limits, upper and lower quartiles; whiskers, 1.5x interquartile range; points, outliers.

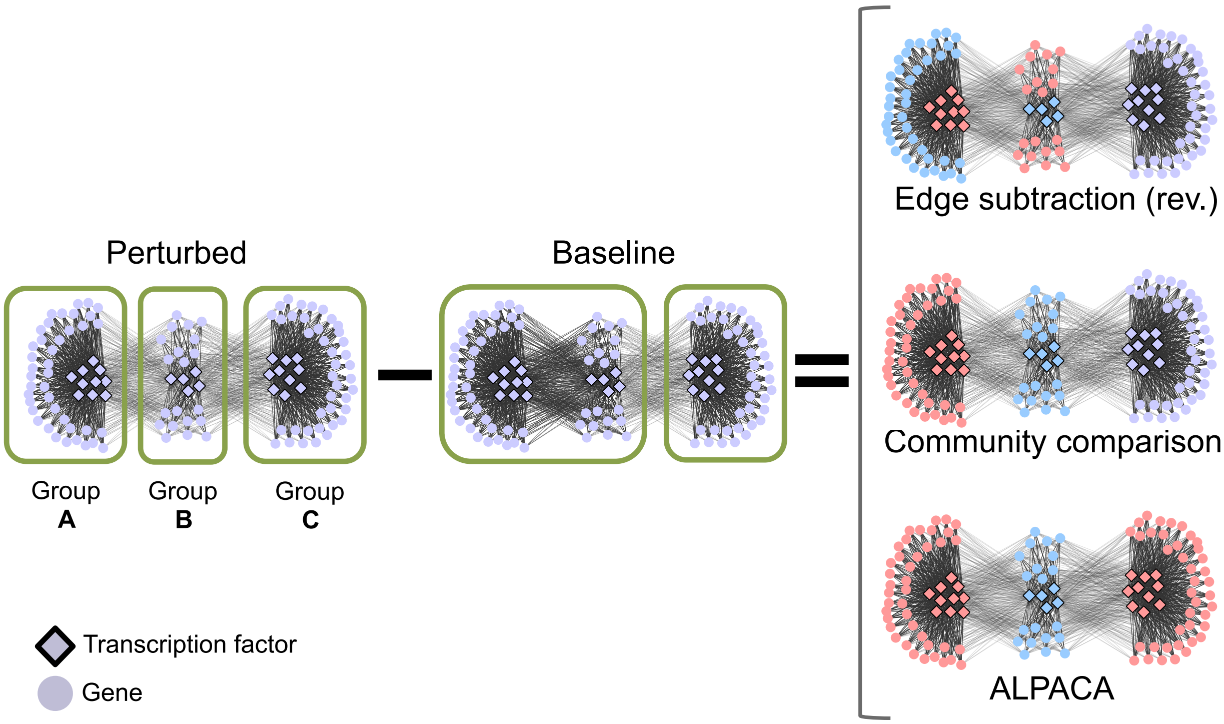

Supplementary Figure 2. Performance of three methods on three-component network with decrease in edge density. Left hand side shows a network transition involving a decrease in edge weights between nodes in Groups A and B. All other edges remain the same. Right hand side shows the results of all three methods when comparing these two networks. Note that the "edge subtraction" method needs to be applied in the reverse manner, comparing the baseline network against the perturbed network, in order to have positive differential edge weights. Each method identified up to two differential modules, which are distinguished by their light blue and light pink colors in each case. The light violet color denotes nodes that remain unclassified by the indicated method.

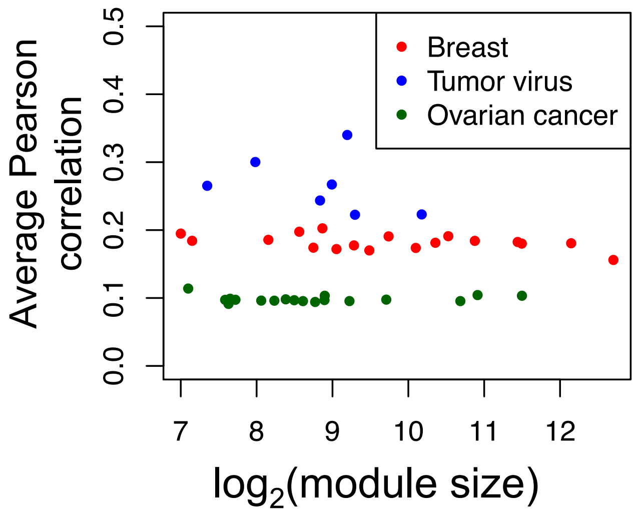

Supplementary Figure 3. Average correlation of gene expression in ALPACA modules. Each point in the scatterplot represents the average of the magnitude of the Pearson correlation coefficient over all gene pairs in one ALPACA module, plotted against the logarithm of the number of genes in the module.
