# Supplementary material for: Detecting phenotype-driven transitions in regulatory network structure
Source: NPJ Syst Biol Appl. 2018 Apr 19;4:16. doi: 10.1038/s41540-018-0052-5 (PMC5908977; doi:10.1038/s41540-018-0052-5)
Supplement: Supplementary file 2 — Supplementary Information (final) [file 41540_2018_52_MOESM2_ESM.pdf]

SUPPLEMENTARY INFORMATION

For "Detecting phenotype-driven transitions in regulatory network structure"

Authors: Megha Padi, John Quackenbush

1. Angiogenic vs. non-angiogenic ovarian cancer tumors

We used ALPACA to compare angiogenic and non-angiogenic ovarian tumors from The Cancer Gene Atlas (TCGA). We examined the resulting modules and their connections to ovarian cancer in detail, focusing on non-redundant GO terms that had an overlap of three genes or more with the module in which it was enriched (Figure 4 and Supplementary Table 1). Module 4 was enriched for "flavonoid glucuronidation" and contains the UDP glucuronosyltransferases UGT2B15, UGT1A8, and UGT2B17, enzymes that can help metabolize flavonoids and regulate hormones. Studies have hinted that dietary intake of flavonoids may reduce the risk of ovarian cancer but the association is not statistically robust, and the mechanism is unknown.[1-3] Our results suggest that the UGT family of enzymes may mediate the connection between flavonoids and ovarian cancer. Module 5 is enriched in "urogenital system development" and contains several genes that are highly relevant to ovarian cancer. HNF1B is known to be a subtype-specific ovarian cancer susceptibility gene.[4] Its expression level and promoter methylation status is predictive of clear cell and invasive serous subtypes of epithelial ovarian cancer. ESR1 is the estrogen receptor and is central to breast and ovarian cancer. IQGAP1 is a scaffold protein whose expression appears to drive invasion and progression of ovarian cancer tumors.[5-7] SOX11 acts as a tumor suppressor in ovarian cancer, and its expression is regulated by methylation and predicts patient survival.[8,9]

We found that module 12 was enriched in triglyceride homeostasis. Although it is not known whether there is a dietary effect of triglycerides on ovarian cancer risk, several studies have noted that ovarian carcinomas have distinctive lipid profiles and metabolic characteristics.[10] Our results suggest that metabolic pathways involving hepatic lipase C (LIPC) and glucokinase regulator (GCKR) may be mobilized differently in poor-prognosis ovarian cancer. Finally, modules 16 and 17 were enriched for various terms involving interferon response, interleukins, and regulation of the NFκB pathway, consistent with the theory that chronic inflammation is associated with risk of cancer.[11] Specifically, the interleukin IL6 has been proposed as a therapeutic target, and IL12 is a prognostic factor in ovarian cancer.[12-14] Interferons have cytotoxic properties in ovarian cancer cells.[15,16] NFκB activation is correlated with poor prognosis in ovarian cancer, and blocking the NFκB pathway can reduce anchorage-independent growth and invasiveness in cell culture assays.[17]

Module 7 was enriched in "blood vessel development" and "positive regulation of cell migration," reflecting the invasive and angiogenic characteristics of poor-prognosis ovarian tumors. The apoptosis gene PDCD6 is a member of both GO terms and is topologically central to this module. Interestingly, it

is a known predictor of progression free survival in ovarian cancer and synergizes with cisplatin to inhibit ovarian cancer cells *in vitro*.[18-20] CYR61, also a member of both GO terms, is an extracellular matrix (ECM) signaling protein that is overexpressed in poor prognosis ovarian carcinoma.[21,22] CTGF (connective tissue growth factor) is an angiogenic ECM protein, and it appears to have an inverse relationship with CYR61; high CTGF expression correlates with low CYR61 in low-grade tumors with increased survival.[23] Overall, this suggests that the ALPACA modules contain functional groups of prognostic genes that may interact with each other to produce distinct phenotypes. ALPACA could therefore be a useful feature selection step to isolate small groups of pathway genes and build more complex predictive models.

Module 7 was also enriched in growth hormones and the JAK-STAT cascade. The JAK-STAT pathway is constitutively active in breast, ovarian and prostate cancers, and nuclear localization of activated STAT3 is associated with worse survival and chemoresistance in ovarian cancer. Treatment with JAK2 inhibitor reduces tumor burden in ovarian cancer xenografts.[24] Members of module 7 that are annotated with this GO term include growth hormones 1 and 2 (GH1 and GH2). This pathway is already known to be a drug target in ovarian cancer, and growth hormone-releasing hormone (GHRH) antagonists reduce proliferation of ovarian cancer cells both *in vitro* and *in vivo*.[25-27]

We ranked all genes in the network by their contribution to the differential modularity, using either the raw score S_i or a normalized version $\tilde{S}_i$ which corrects for the size of the module, and then performed Gene Set Enrichment Analysis (GSEA) (see Materials and Methods for more details). The normalized version of the score resulted in much stronger functional enrichment, including interferon response, interleukin secretion, blood vessel remodeling, and epithelial cell apoptosis (Supplementary Table 4). Many of the significant GSEA results were also found in the module-specific analysis described above.

Among the differentially expressed genes (DEGs) between non-angiogenic and angiogenic tumors, we found strong enrichment for “blood vessel development” and “angiogenesis,” as well as processes related to cell migration, cell adhesion, and embryonic morphogenesis (Supplementary Table 1). The DEGs were not enriched for any of the other functions found using ALPACA, like interferon response, interleukin production, or flavonoid biosynthesis. Moreover, we found that only about half of the GO term-associated genes and core transcription factors in the ALPACA modules were differentially expressed with $P_{adj} < 0.05$. In other words, the ALPACA modules do not merely recapitulate the top differentially expressed genes and regulators.

2. Tumor virus perturbations in primary human cells

We compared the regulatory network active in cells expressing transforming viral oncogenes with the network active in cells expressing control vectors. ALPACA was the only method to identify communities representing several cancer pathways that are known to be targeted by tumor viruses,

including extracellular matrix (ECM) organization, NFκB signaling, and embryonic development (Figure 5 and Supplementary Table 2). Module 1 is enriched in "cellular calcium ion homeostasis" and "regulation of NIK/NF-kappaB signaling." NFκB and Nuclear factor of activated T-cells (NFAT) are two important cancer-related pathways that activate immune cells, and NFAT activity is modulated primarily through intracellular calcium levels. Merkel cell polyomavirus and EBV LMP1 are both known to functionally perturb the NFκB pathway through different mechanisms.[28,29] EBV, HPV16 and several polyomaviruses target the genes CHI3L1, TLR9, and SOCS1, which are all among the top-scoring nodes in this module.[30-35] EBV and HPV infections both alter calcium signaling in the host cell.[36,37] Tumor viruses use these pathways in a variety of ways to increase cell growth and manipulate the innate immune response.

We found that module 4 was enriched in many terms related to "embryonic morphogenesis" and development. We previously found that tumor viruses co-opt the Notch pathway, which is central to embryonic development, in order to promote cell growth and tumorigenesis.[38] The GO term enrichment among the target genes in module 4 is driven by the homeobox (HOX) TFs, whose expression is regulated by EBV LMP1 and HPV E7 through differential methylation.[39-41] Module 4 was also enriched in "extracellular matrix organization." The epithelial-to-mesenchymal (EMT) transition is a key step in epithelial tumorigenesis, and cells undergoing EMT often acquire the ability to degrade extracellular matrix (ECM) proteins and increase their invasive potential.[42] In particular, the transforming HPV E6 and E7 proteins are able to upregulate matrix metalloproteinases (MMPs) in order to degrade ECM and increase cell migration, thus leading to cellular transformation.[43]

Both ALPACA and the edge subtraction method detected a difference in the regulation of histones, suggesting that epigenetic changes may be a key factor in the transformation of human cells by viral oncogenes. ALPACA also identified a separate module (module 8) that was enriched in proteins involved in "DNA conformation change." Indeed, the importance of epigenetics in transformation has already been demonstrated for many tumor viruses. HPV16 E7 induces histone 3 lysine 27-specific demethylases,[41] EBV LMP1 and LMP2A modulate the activity of DNA methyltransferases and interact with histone modifiers,[44] and adenovirus E1A causes sweeping changes in histone acetylation.[45]

Ranking all the genes by their contribution to the differential modularity and running GSEA resulted in a number of significant pathways related to chromatin silencing, cell differentiation, chemotaxis and morphogenesis (Supplementary Table 4).

We also studied the differentially expressed genes between cell lines expressing transforming viral proteins and control cell lines. The DEGs were enriched in GO terms pertaining to interferon response, negative regulation of cell death, cell communication and NFκB activity, but there was no enrichment in many of the GO terms found using ALPACA, including mitochondrial translation, mitotic cell cycle, DNA conformation change, and chromatin assembly (Supplementary Table 2). The top 100

DEGs were enriched for “negative regulation of cell death,” but the genes in this category had no overlap with the genes annotated with “mitotic cell cycle.” Only 4 of the 23 genes annotated with “mitotic cell cycle” were significantly differentially expressed. None of the transcription factors regulating the mitotic cell cycle module - RB1, TFDP1, ATF1, ATF2 and ATF6 – were differentially expressed.

3. Sexual dimorphism in normal breast tissue

We tested whether ALPACA could find sex-specific modular structure in the breast regulatory network (Figure 6). We first compared the male regulatory network against the female regulatory network and found 18 male-specific differential modules (Supplementary Table 3). Module 2 was highly enriched in developmental processes, including “nervous system development,” “response to BMP,” and “blood vessel development.” Similarly, module 8 was enriched for “muscle organ morphogenesis.” These results are not surprising and reflect the fact that male and female breast tissues have significant differences in their developmental trajectory. We note that many of the developmental genes in these modules are associated with breast cancer. Among genes annotated with “nervous system development,” the fibroblast growth factor receptor (FGFR) is often amplified or dysregulated in breast cancer, the HES5 locus is repositioned in invasive breast cancer, and VLDLR is often upregulated in metastatic breast cancer.[46-49] The blood vessel development category included genes such as GATA6, a known oncogene that may drive EMT in the breast; TBX3, which appears to repress the tumor suppressor p14ARF and drive metastatic breast cancer; PRRX2, which increases invasiveness in breast tumors; and RASA1, whose expression is associated with poor prognosis in breast cancer.[50-53] Among the BMP response and muscle development groups, there are several genes, like TWSG1, VANGL2, and GSC, which are relevant to both normal breast development and breast cancer.[54-56] Module 7 was enriched for terms related to rRNA processing and module 14 contained genes relevant to chromatin assembly, suggesting that transcription and translation are reorganized at a global level between males and females.

Next, we compared the female breast regulatory network against the male network and found 17 female-specific regulatory modules. Among those, module 15 is enriched in “intracellular estrogen receptor signaling pathway,” a pathway that is known to be critical for female breast development. The highest-scoring gene in this pathway, PPARGC1B, is a co-activator of the estrogen receptor and is a genetic risk factor for estrogen receptor-positive breast cancer.[57,58] Module 10 was enriched for “positive regulation of ERK1 and ERK2 cascade.” ERK1/2 signaling is a major pathway involved in estrogen-induced cell proliferation and breast cancer.[59,60] This module contains the growth arrest-specific gene GAS6, which is induced by estrogen and is associated with chemoresistance and metastasis in breast cancer,[61-63] and the chemokine CCL5, which has been proposed as a therapeutic target for estrogen-dependent breast cancer.[64,65] Module 10 was also enriched for Type I interferon response, which may be a result of the increased blood and lymphatic penetrance in normal female

breast development. We found module 17 to be enriched for "negative regulation of cell-substrate adhesion" and contained SPOCK1 and NOTCH1, both known markers of invasion and breast cancer progression.[66-69] Finally, module 5 was enriched in transcriptional regulation factors, similar to the enrichment in chromatin remodeling found in the male breast network.

Applying GSEA to all the genes ranked by their contribution to the differential modularity in female breast tissue, we found enrichment for mitosis and DNA replication, Notch signaling, and chromatin organization (Supplementary Table 4). Intracellular estrogen receptor signaling was positively enriched with an FDR of 0.254, which was slightly too high to pass the GSEA significance threshold. In male breast tissue, the most significantly enriched pathways related to chromatin modification, cell-cell junctions, and differentiation pathways.

In contrast, the genes that are differentially expressed between male and female breast tissue are enriched for processes like immune response, protein localization, chemotaxis, MAPKK activity, and DNA damage response (Supplementary Table 3). The differentially expressed genes are not enriched for estrogen receptor signaling, GTPase activity, ERK1/2 cascade, and many other processes that were identified through ALPACA analysis, regardless of the significance threshold used to define differential expression. In general, we found that most of the core genes in the ALPACA modules are not differentially expressed. For example, only one of the genes annotated to the estrogen receptor signaling pathway is differentially expressed.

In summary, consistent with expectations based on both the functional differences of male and female breast, and the profound differences in gene expression, ALPACA was able to identify major biological processes associated with differences in breast development between females and males, many of which are also known to be dysregulated in breast cancer.

REFERENCES

1. Cassidy, A., Huang, T., Rice, M.S., Rimm, E.B. & Tworoger, S.S. Intake of dietary flavonoids and risk of epithelial ovarian cancer. *The American journal of clinical nutrition* **100**, 1344-1351 (2014).
2. Gates, M.A., Vitonis, A.F., Tworoger, S.S., Rosner, B., Titus-Ernstoff, L.*, et al.* Flavonoid intake and ovarian cancer risk in a population-based case-control study. *International journal of cancer* **124**, 1918-1925 (2009).
3. Hua, X., Yu, L., You, R., Yang, Y., Liao, J.*, et al.* Association among Dietary Flavonoids, Flavonoid Subclasses and Ovarian Cancer Risk: A Meta-Analysis. *PloS one* **11**, e0151134 (2016).
4. Shen, H., Fridley, B.L., Song, H., Lawrenson, K., Cunningham, J.M.*, et al.* Epigenetic analysis leads to identification of HNF1B as a subtype-specific susceptibility gene for ovarian cancer. *Nature communications* **4**, 1628 (2013).
5. Bourguignon, L.Y., Gilad, E., Rothman, K. & Peyrollier, K. Hyaluronan-CD44 interaction with IQGAP1 promotes Cdc42 and ERK signaling, leading to actin binding, Elk-1/estrogen receptor transcriptional activation, and ovarian cancer progression. *The Journal of biological chemistry* **280**, 11961-11972 (2005).

6. Dong, P., Nabeshima, K., Nishimura, N., Kawakami, T., Hachisuga, T.*, et al.* Overexpression and diffuse expression pattern of IQGAP1 at invasion fronts are independent prognostic parameters in ovarian carcinomas. *Cancer letters* **243**, 120-127 (2006).
7. Dong, P.X., Jia, N., Xu, Z.J., Liu, Y.T., Li, D.J.*, et al.* Silencing of IQGAP1 by shRNA inhibits the invasion of ovarian carcinoma HO-8910PM cells in vitro. *Journal of experimental & clinical cancer research : CR* **27**, 77 (2008).
8. Brennan, D.J., Ek, S., Doyle, E., Drew, T., Foley, M.*, et al.* The transcription factor Sox11 is a prognostic factor for improved recurrence-free survival in epithelial ovarian cancer. *European journal of cancer (Oxford, England : 1990)* **45**, 1510-1517 (2009).
9. Sernbo, S., Gustavsson, E., Brennan, D.J., Gallagher, W.M., Rexhepaj, E.*, et al.* The tumour suppressor SOX11 is associated with improved survival among high grade epithelial ovarian cancers and is regulated by reversible promoter methylation. *BMC cancer* **11**, 405 (2011).
10. Tania, M., Khan, M.A. & Song, Y. Association of lipid metabolism with ovarian cancer. *Current oncology (Toronto, Ont.)* **17**, 6-11 (2010).
11. Hanahan, D. & Weinberg, R.A. Hallmarks of cancer: the next generation. *Cell* **144**, 646-674 (2011).
12. Cohen, C.A., Shea, A.A., Heffron, C.L., Schmelz, E.M. & Roberts, P.C. Interleukin-12 Immunomodulation Delays the Onset of Lethal Peritoneal Disease of Ovarian Cancer. *Journal of interferon & cytokine research : the official journal of the International Society for Interferon and Cytokine Research* **36**, 62-73 (2016).
13. Coward, J., Kulbe, H., Chakravarty, P., Leader, D., Vassileva, V.*, et al.* Interleukin-6 as a therapeutic target in human ovarian cancer. *Clinical cancer research : an official journal of the American Association for Cancer Research* **17**, 6083-6096 (2011).
14. Isobe, A., Sawada, K., Kinose, Y., Ohyagi-Hara, C., Nakatsuka, E.*, et al.* Interleukin 6 receptor is an independent prognostic factor and a potential therapeutic target of ovarian cancer. *PloS one* **10**, e0118080 (2015).
15. Wall, L., Burke, F., Barton, C., Smyth, J. & Balkwill, F. IFN-gamma induces apoptosis in ovarian cancer cells in vivo and in vitro. *Clinical cancer research : an official journal of the American Association for Cancer Research* **9**, 2487-2496 (2003).
16. Welander, C.E. Use of interferon in the treatment of ovarian cancer as a single agent and in combination with cytotoxic drugs. *Cancer* **59**, 617-619 (1987).
17. Alvero, A.B. Recent insights into the role of NF-kappaB in ovarian carcinogenesis. *Genome medicine* **2**, 56 (2010).
18. Huang, Y., Jin, H., Liu, Y., Zhou, J., Ding, J.*, et al.* FSH inhibits ovarian cancer cell apoptosis by up-regulating survivin and down-regulating PDCD6 and DR5. *Endocrine-related cancer* **18**, 13-26 (2011).
19. Park, S.H., Lee, J.H., Lee, G.B., Byun, H.J., Kim, B.R.*, et al.* PDCD6 additively cooperates with anti-cancer drugs through activation of NF-kappaB pathways. *Cellular signalling* **24**, 726-733 (2012).
20. Su, D., Xu, H., Feng, J., Gao, Y., Gu, L.*, et al.* PDCD6 is an independent predictor of progression free survival in epithelial ovarian cancer. *Journal of translational medicine* **10**, 31 (2012).
21. Lin, Y., Xu, T., Tian, G. & Cui, M. Cysteine-rich, angiogenic inducer, 61 expression in patients with ovarian epithelial carcinoma. *The Journal of international medical research* **42**, 300-306 (2014).

22. Shen, H., Cai, M., Zhao, S., Wang, H., Li, M., *et al.* CYR61 overexpression associated with the development and poor prognosis of ovarian carcinoma. *Medical oncology (Northwood, London, England)* **31**, 117 (2014).
23. Bartel, F., Balschun, K., Gradhand, E., Strauss, H.G., Dittmer, J., *et al.* Inverse expression of cystein-rich 61 (Cyr61/CCN1) and connective tissue growth factor (CTGF/CCN2) in borderline tumors and carcinomas of the ovary. *International journal of gynecological pathology : official journal of the International Society of Gynecological Pathologists* **31**, 405-415 (2012).
24. Abubaker, K., Luwor, R.B., Zhu, H., McNally, O., Quinn, M.A., *et al.* Inhibition of the JAK2/STAT3 pathway in ovarian cancer results in the loss of cancer stem cell-like characteristics and a reduced tumor burden. *BMC cancer* **14**, 317 (2014).
25. Guo, J., Schally, A.V., Zarandi, M., Varga, J. & Leung, P.C. Antiproliferative effect of growth hormone-releasing hormone (GHRH) antagonist on ovarian cancer cells through the EGFR-Akt pathway. *Reproductive biology and endocrinology : RB&E* **8**, 54 (2010).
26. Klukovits, A., Schally, A.V., Szalontay, L., Vidaurre, I., Papadia, A., *et al.* Novel antagonists of growth hormone-releasing hormone inhibit growth and vascularization of human experimental ovarian cancers. *Cancer* **118**, 670-680 (2012).
27. Papadia, A., Schally, A.V., Halmos, G., Varga, J.L., Seitz, S., *et al.* Growth hormone-releasing hormone antagonists inhibit growth of human ovarian cancer. *Hormone and metabolic research = Hormon- und Stoffwechselforschung = Hormones et metabolisme* **43**, 816-820 (2011).
28. Griffiths, D.A., Abdul-Sada, H., Knight, L.M., Jackson, B.R., Richards, K., *et al.* Merkel cell polyomavirus small T antigen targets the NEMO adaptor protein to disrupt inflammatory signaling. *Journal of virology* **87**, 13853-13867 (2013).
29. Hiscott, J., Kwon, H. & Genin, P. Hostile takeovers: viral appropriation of the NF-kappaB pathway. *The Journal of clinical investigation* **107**, 143-151 (2001).
30. Auburn, H., Zuckerman, M. & Smith, M. Analysis of Epstein-Barr virus and cellular gene expression during the early phases of EBV lytic induction. *Journal of medical microbiology* (2016).
31. Hasan, U.A., Bates, E., Takeshita, F., Biliato, A., Accardi, R., *et al.* TLR9 expression and function is abolished by the cervical cancer-associated human papillomavirus type 16. *Journal of immunology (Baltimore, Md. : 1950)* **178**, 3186-3197 (2007).
32. Shahzad, N., Shuda, M., Gheit, T., Kwun, H.J., Cornet, I., *et al.* The T antigen locus of Merkel cell polyomavirus downregulates human Toll-like receptor 9 expression. *Journal of virology* **87**, 13009-13019 (2013).
33. Zauner, L. & Nadal, D. Understanding TLR9 action in Epstein-Barr virus infection. *Frontiers in bioscience (Landmark edition)* **17**, 1219-1231 (2012).
34. Assetta, B., De Cecco, M., O'Hara, B. & Atwood, W.J. JC Polyomavirus Infection of Primary Human Renal Epithelial Cells Is Controlled by a Type I IFN-Induced Response. *mBio* **7**(2016).
35. Michaud, F., Coulombe, F., Gaudreault, E., Paquet-Bouchard, C., Rola-Pleszczynski, M., *et al.* Epstein-Barr virus interferes with the amplification of IFNalpha secretion by activating suppressor of cytokine signaling 3 in primary human monocytes. *PloS one* **5**, e11908 (2010).
36. Turunen, A. & Syrjanen, S. Extracellular calcium regulates keratinocyte proliferation and HPV 16 E6 RNA expression in vitro. *APMIS : acta pathologica, microbiologica, et immunologica Scandinavica* **122**, 781-789 (2014).
37. Chami, M., Oules, B. & Paterlini-Brechot, P. Cytobiological consequences of calcium-signaling alterations induced by human viral proteins. *Biochimica et biophysica acta* **1763**, 1344-1362 (2006).

38. Rozenblatt-Rosen, O., Deo, R.C., Padi, M., Adelmant, G., Calderwood, M.A., *et al.* Interpreting cancer genomes using systematic host network perturbations by tumour virus proteins. *Nature* **487**, 491-495 (2012).
39. Hernando, H., Islam, A.B., Rodriguez-Ubreva, J., Forne, I., Ciudad, L., *et al.* Epstein-Barr virus-mediated transformation of B cells induces global chromatin changes independent to the acquisition of proliferation. *Nucleic acids research* **42**, 249-263 (2014).
40. Jiang, Y., Yan, B., Lai, W., Shi, Y., Xiao, D., *et al.* Repression of Hox genes by LMP1 in nasopharyngeal carcinoma and modulation of glycolytic pathway genes by HoxC8. *Oncogene* **34**, 6079-6091 (2015).
41. McLaughlin-Drubin, M.E., Crum, C.P. & Munger, K. Human papillomavirus E7 oncoprotein induces KDM6A and KDM6B histone demethylase expression and causes epigenetic reprogramming. *Proceedings of the National Academy of Sciences of the United States of America* **108**, 2130-2135 (2011).
42. Lamouille, S., Xu, J. & Derynck, R. Molecular mechanisms of epithelial-mesenchymal transition. *Nature reviews. Molecular cell biology* **15**, 178-196 (2014).
43. Zhu, D., Ye, M. & Zhang, W. E6/E7 oncoproteins of high risk HPV-16 upregulate MT1-MMP, MMP-2 and MMP-9 and promote the migration of cervical cancer cells. *International journal of clinical and experimental pathology* **8**, 4981-4989 (2015).
44. Niller, H.H., Szenthe, K. & Minarovits, J. Epstein-Barr virus-host cell interactions: an epigenetic dialog? *Frontiers in genetics* **5**, 367 (2014).
45. Ferrari, R., Pellegrini, M., Horwitz, G.A., Xie, W., Berk, A.J., *et al.* Epigenetic reprogramming by adenovirus e1a. *Science (New York, N.Y.)* **321**, 1086-1088 (2008).
46. He, L., Lu, Y., Wang, P., Zhang, J., Yin, C., *et al.* Up-regulated expression of type II very low density lipoprotein receptor correlates with cancer metastasis and has a potential link to beta-catenin in different cancers. *BMC cancer* **10**, 601 (2010).
47. Meaburn, K.J., Gudla, P.R., Khan, S., Lockett, S.J. & Misteli, T. Disease-specific gene repositioning in breast cancer. *The Journal of cell biology* **187**, 801-812 (2009).
48. Turner, N. & Grose, R. Fibroblast growth factor signalling: from development to cancer. *Nature reviews. Cancer* **10**, 116-129 (2010).
49. Webb, D.J., Nguyen, D.H., Sankovic, M. & Gonias, S.L. The very low density lipoprotein receptor regulates urokinase receptor catabolism and breast cancer cell motility in vitro. *The Journal of biological chemistry* **274**, 7412-7420 (1999).
50. Krstic, M., Macmillan, C.D., Leong, H.S., Clifford, A.G., Souter, L.H., *et al.* The transcriptional regulator TBX3 promotes progression from non-invasive to invasive breast cancer. *BMC cancer* **16**, 671 (2016).
51. Okada, T., Sinha, S., Esposito, I., Schiavon, G., Lopez-Lago, M.A., *et al.* The Rho GTPase Rnd1 suppresses mammary tumorigenesis and EMT by restraining Ras-MAPK signalling. *Nature cell biology* **17**, 81-94 (2015).
52. Song, Y., Tian, T., Fu, X., Wang, W., Li, S., *et al.* GATA6 is overexpressed in breast cancer and promotes breast cancer cell epithelial-mesenchymal transition by upregulating slug expression. *Experimental and molecular pathology* **99**, 617-627 (2015).
53. Yarosh, W., Barrientos, T., Esmailpour, T., Lin, L., Carpenter, P.M., *et al.* TBX3 is overexpressed in breast cancer and represses p14 ARF by interacting with histone deacetylases. *Cancer research* **68**, 693-699 (2008).
54. Forsman, C.L., Ng, B.C., Heinze, R.K., Kuo, C., Sergi, C., *et al.* BMP-binding protein twisted gastrulation is required in mammary gland epithelium for normal ductal elongation and myoepithelial compartmentalization. *Developmental biology* **373**, 95-106 (2013).

55. Hartwell, K.A., Muir, B., Reinhardt, F., Carpenter, A.E., Sgroi, D.C., *et al.* The Spemann organizer gene, Goosecoid, promotes tumor metastasis. *Proceedings of the National Academy of Sciences of the United States of America* **103**, 18969-18974 (2006).
56. Puvirajesinghe, T.M., Bertucci, F., Jain, A., Scerbo, P., Belotti, E., *et al.* Identification of p62/SQSTM1 as a component of non-canonical Wnt VANGL2-JNK signalling in breast cancer. *Nature communications* **7**, 10318 (2016).
57. Li, Y., Li, Y., Wedren, S., Li, G., Charn, T.H., *et al.* Genetic variation of ESR1 and its co-activator PPARGC1B is synergistic in augmenting the risk of estrogen receptor-positive breast cancer. *Breast cancer research : BCR* **13**, R10 (2011).
58. Wirtenberger, M., Tchatchou, S., Hemminki, K., Schmutzhard, J., Sutter, C., *et al.* Associations of genetic variants in the estrogen receptor coactivators PPARGC1A, PPARGC1B and EP300 with familial breast cancer. *Carcinogenesis* **27**, 2201-2208 (2006).
59. Jerjees, D.A., Alabdullah, M., Alkaabi, M., Abduljabbar, R., Muftah, A., *et al.* ERK1/2 is related to oestrogen receptor and predicts outcome in hormone-treated breast cancer. *Breast cancer research and treatment* **147**, 25-37 (2014).
60. Keshamouni, V.G., Mattingly, R.R. & Reddy, K.B. Mechanism of 17-beta-estradiol-induced Erk1/2 activation in breast cancer cells. A role for HER2 AND PKC-delta. *The Journal of biological chemistry* **277**, 22558-22565 (2002).
61. Mc Cormack, O., Chung, W.Y., Fitzpatrick, P., Cooke, F., Flynn, B., *et al.* Growth arrest-specific gene 6 expression in human breast cancer. *British journal of cancer* **98**, 1141-1146 (2008).
62. Mo, R., Tony Zhu, Y., Zhang, Z., Rao, S.M. & Zhu, Y.J. GAS6 is an estrogen-inducible gene in mammary epithelial cells. *Biochemical and biophysical research communications* **353**, 189-194 (2007).
63. Wang, C., Jin, H., Wang, N., Fan, S., Wang, Y., *et al.* Gas6/Axl Axis Contributes to Chemoresistance and Metastasis in Breast Cancer through Akt/GSK-3beta/beta-catenin Signaling. *Theranostics* **6**, 1205-1219 (2016).
64. Soria, G. & Ben-Baruch, A. The inflammatory chemokines CCL2 and CCL5 in breast cancer. *Cancer letters* **267**, 271-285 (2008).
65. Svensson, S., Abrahamsson, A., Rodriguez, G.V., Olsson, A.K., Jensen, L., *et al.* CCL2 and CCL5 Are Novel Therapeutic Targets for Estrogen-Dependent Breast Cancer. *Clinical cancer research : an official journal of the American Association for Cancer Research* **21**, 3794-3805 (2015).
66. Bolos, V., Mira, E., Martinez-Poveda, B., Luxan, G., Canamero, M., *et al.* Notch activation stimulates migration of breast cancer cells and promotes tumor growth. *Breast cancer research : BCR* **15**, R54 (2013).
67. Fan, L.C., Jeng, Y.M., Lu, Y.T. & Lien, H.C. SPOCK1 Is a Novel Transforming Growth Factor-beta-Induced Myoepithelial Marker That Enhances Invasion and Correlates with Poor Prognosis in Breast Cancer. *PloS one* **11**, e0162933 (2016).
68. Simmons, M.J., Serra, R., Hermance, N. & Kelliher, M.A. NOTCH1 inhibition in vivo results in mammary tumor regression and reduced mammary tumorsphere-forming activity in vitro. *Breast cancer research : BCR* **14**, R126 (2012).
69. Wang, J., Fu, L., Gu, F. & Ma, Y. Notch1 is involved in migration and invasion of human breast cancer cells. *Oncology reports* **26**, 1295-1303 (2011).
